# Supplementary material for: Non-invasive hydrodynamic imaging in plant roots at cellular resolution
Source: Nat Commun. 2021 Aug 3;12:4682. doi: 10.1038/s41467-021-24913-z (PMC8333316; doi:10.1038/s41467-021-24913-z)
Supplement: Supplementary file 1 — Supplementary Information file [file 41467_2021_24913_MOESM1_ESM.pdf]

Non-invasive hydrodynamic imaging in plant roots at cellular resolution

Pascut *et al.*

Supplementary Table 1. Types of hydrophobic barriers along the primary roots of *Arabidopsis thaliana* lines used in this study.

**Position** / **Line**	**0 – 3 mm**	**3 – 10.5 mm**	**10.5 mm – proximal end**
WT	Casparian strip	Casparian strip	Casparian strip and suberin lamellae
*esb1-1*CDEF*	No barrier	Defective Casparian strip and ectopic lignin	Defective Casparian strip and ectopic lignin
CDEF	Casparian strip	Casparian strip	Casparian strip
sgn3-3 myb36-2	No barrier	No barrier	Suberin lamellae

Position zero corresponds to the maturation point of protoxylem vessels, and higher positions are located closer to the shoot. The Casparian strip blocks apoplastic flow in radial cell walls of the endodermis. The suberin lamellae block transmembrane flow between apoplast and symplast of suberized cells.

Supplementary Table 2. Optimized parameter values obtained using inverse modelling of root hydraulic conductivity data (for the 3 hydraulic parameters: k_w, K_{PD} and k_{AQP}) and D_2O wash-out traces (for the 3 diffusion parameters and xylem water flow rates: D_w, D_{PD}, D_x and Q_{xyl}).

**Parameter (units)**	**Optimized parameter values (one value of Q_{xyl} per replicate trace)**
k_w ($m^2\ MPa^{-1}\ s^{-1}$)	2.5 10^{-12}
K_{PD} ($m^3\ MPa^{-1}\ s^{-1}$)	1.1 10^{-19}
k_{AQP} ($m\ \ MPa^{-1}\ s^{-1}$)	5.4 10^{-7}
D_w ($m^2\ s^{-1}$)	9.4 10^{-10}
D_{PD} ($m^2\ s^{-1}$)	8.9 10^{-10}
D_x ($m^2\ s^{-1}$)	1.2 10^{-9}
Q_{xyl} ($m^3\ s^{-1}$) x 10^{14}<br>Line: WT	9.8; 10.8; 14.2; 10.2, 9.6; 10.3; 6.5; 9.3; 10.1; 8.8; 11.8; 7.2; 9.4; 12.3; 12.5; 7.5; 8.4; 6.6; 9.3; 8.7; 8.0; 9.5; 8.8; 8.7;
Q_{xyl} ($m^3\ s^{-1}$) x 10^{14}<br>Line: *CDEF*	14.8; 15.2; 16.6; 14.1; 18.9; 17.1; 14.9; 17.6; 17.5; 18.6; 14.8; 16.1; 13.7; 14.2; 19.9; 20.5; 18.9; 14.4;
Q_{xyl} ($m^3\ s^{-1}$) x 10^{14}<br>Line: *sgn3 myb36*	22.3; 11.7; 14.0; 17.0; 12.4; 13.3; 22.2; 17.5; 12.7;

Supplementary Table 3. Plasmodesmata (PD) frequencies per unit plasma membrane surface within or at transitions between *Arabidopsis thaliana* root tissues, as measured by Zhu *et al*. Unavailable values for tissues or transitions between tissues are set to the average value of 0.35 PD μm^{-2}.

**Tissue (or transition)**	**Frequency (PD μm^{-2})**
Epidermis - cortex	0.13
Cortex - cortex	0.51
Cortex - endodermis	0.26
Endodermis - endodermis	0.31
Endodermis - pericycle	0.30
Companion cell – phloem sieve tube	0.90
Other tissues or transitions	0.35

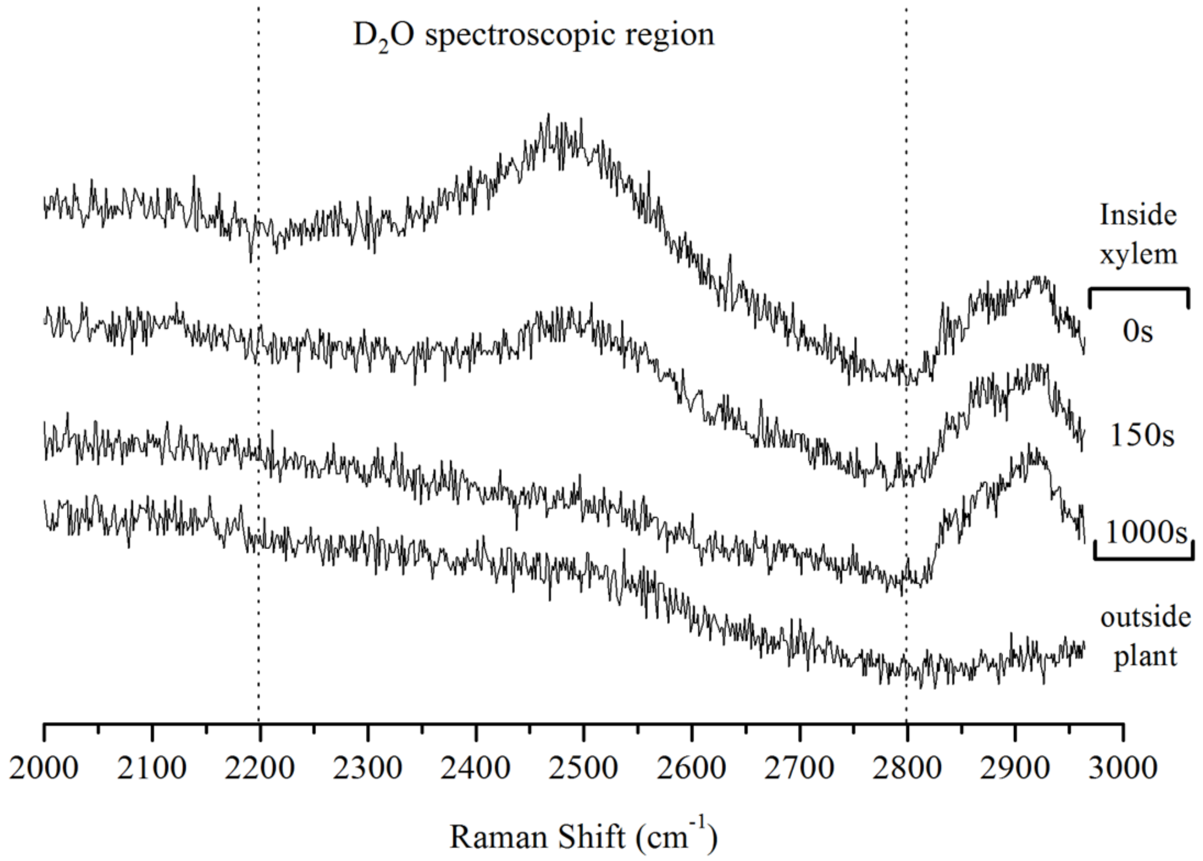

Supplementary Figure 1. Typical Raman spectra for D_2O wash-out, obtained outside the plant (for baseline correction), and from within the xylem at t=0s, t=150s, and 1000s (the end of measurement). Each Raman spectrum can be divided into three regions: 2000-2200cm^{-1} contains no relevant features, therefore is not used in the analysis. 2200-2800cm^{-1} is the D_2O spectroscopic region - in this part of the Raman spectrum only D_2O contributes to the signal, which is void of any Raman-active plant-specific features. The region between 2800cm^{-1} and higher contains features from plant constituents, with lipids and water features dominating this region.

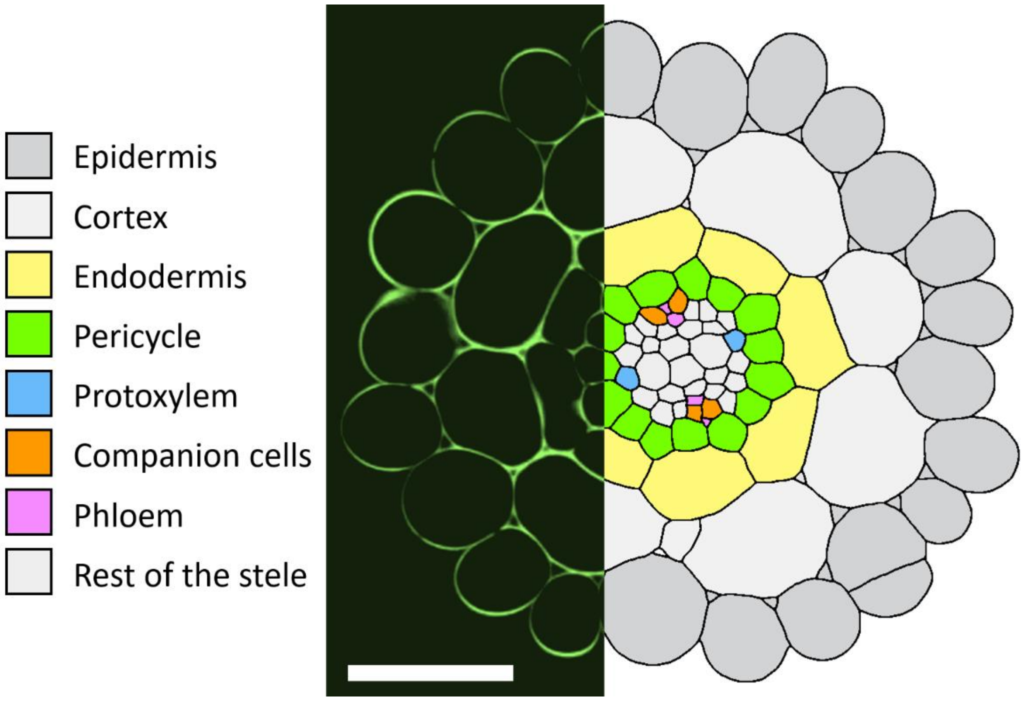

Supplementary Figure 2. *A. thaliana* root anatomy digitized with the software CellSet. Different colors in the legend highlight different cell types. Bar = 25 µm.

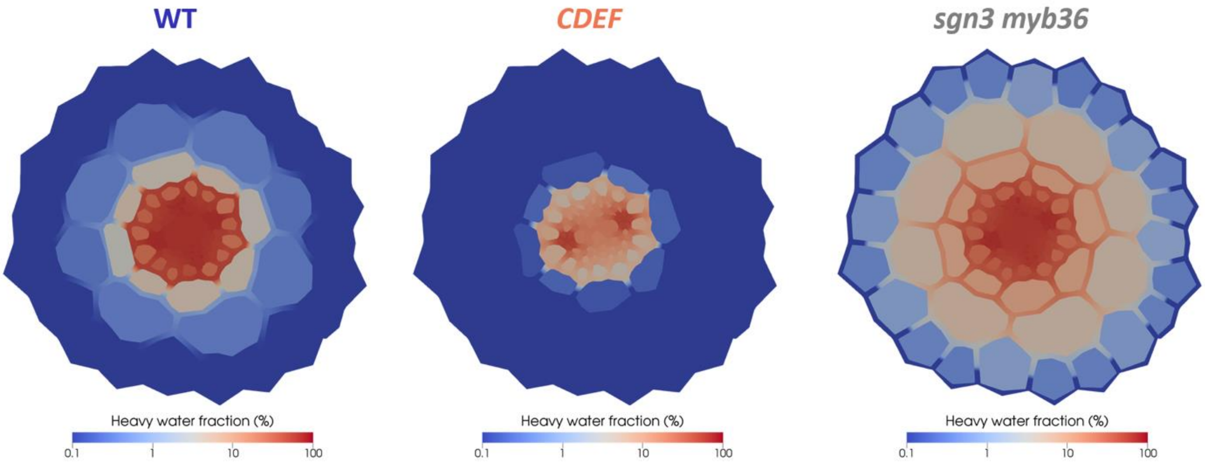

Supplementary Figure 3. Exemplar simulated snapshots of D_2O distribution at the laser focal point before the start of the wash-out phase in *A. thaliana* wild-type (WT) and endodermal barrier mutants (*CDEF* and *sgn3 myb36*). WT takes up little water in the proximal region due to its endodermal suberin lamellae, such that the balance between inner convection and outer diffusion through plasmodesmata allows less than 1% of D_2O to diffuse to the cortex. In contrast, the inward convection rate is higher in the *CDEF* proximal region due to the absence of suberin, thus limiting the outward diffusion of D_2O. Due to the absence of Casparian strip in *sgn3 myb36*, D_2O may diffuse toward the cortex through cell walls.

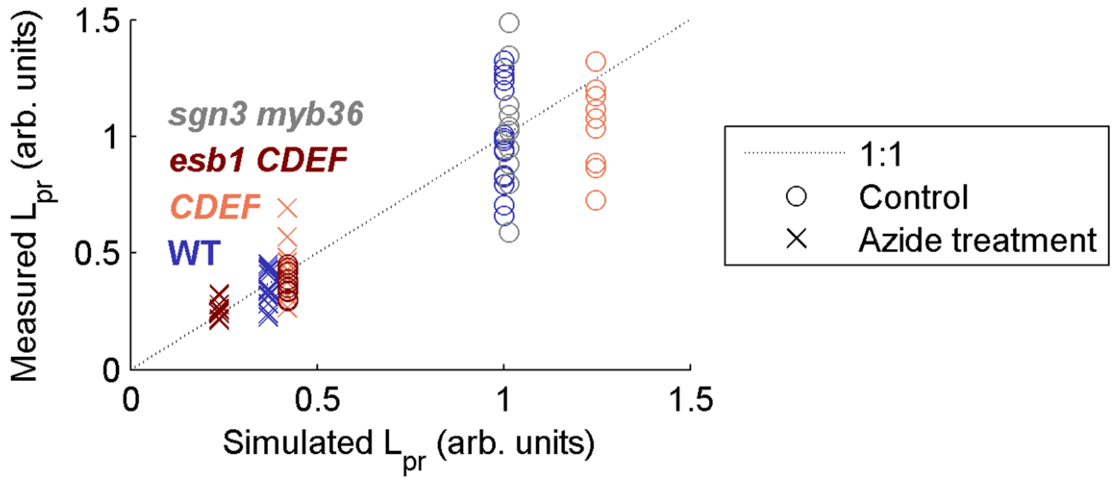

Supplementary Figure 4. Fit of measured and simulated root hydraulic conductivities, used to constrain the cell hydraulic parameters (WT: N=15; *CDEF*: N=12; *esb1 CDEF*: N=10; *sgn3 myb36*: N=10 independent plants). The individual measurements correspond to distributions shown in Fig. 2E.

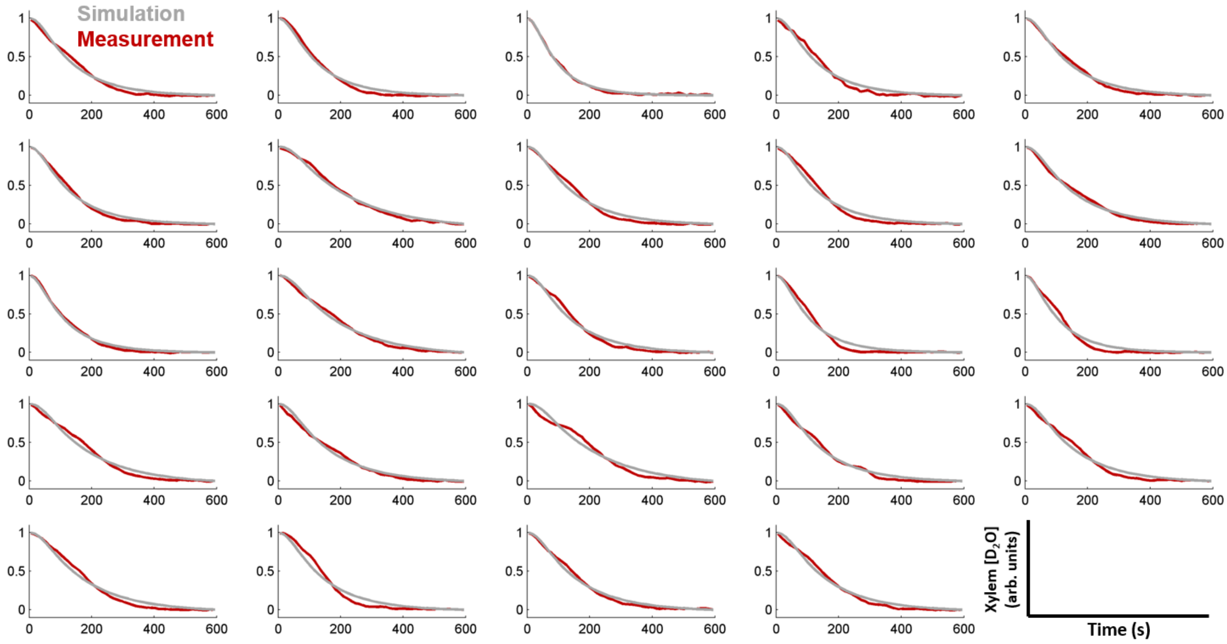

Supplementary Figure 5. Normalized xylem D_2O wash-out traces measured experimentally (red) and fitted by inverse modelling (gray) in wild-type *Arabidopsis thaliana*. Note that axes labels are given at the bottom-right corner of the figure.

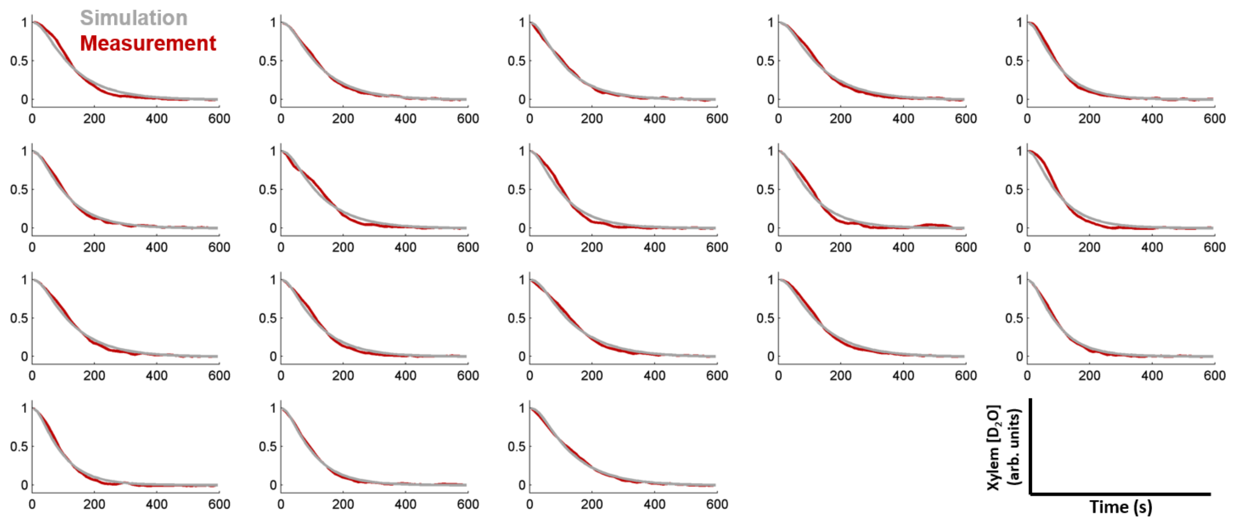

Supplementary Figure 6. Normalized xylem D_2O wash-out traces measured experimentally (red) and fitted by inverse modelling (gray) in *Arabidopsis thaliana* lines ectopically expressing *CDEF*. Note that axes labels are given at the bottom-right corner of the figure.

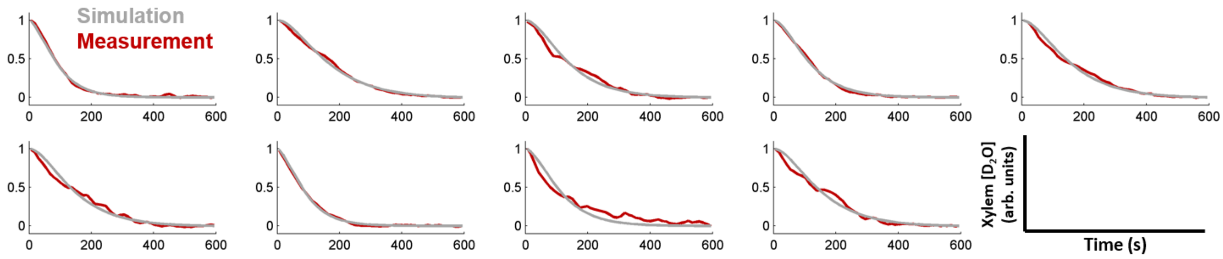

Supplementary Figure 7. Normalized xylem D_2O wash-out traces measured experimentally (red) and fitted by inverse modelling (gray) in *Arabidopsis thaliana sgn3 myb36* mutants. Note that axes labels are given at the bottom-right corner of the figure.

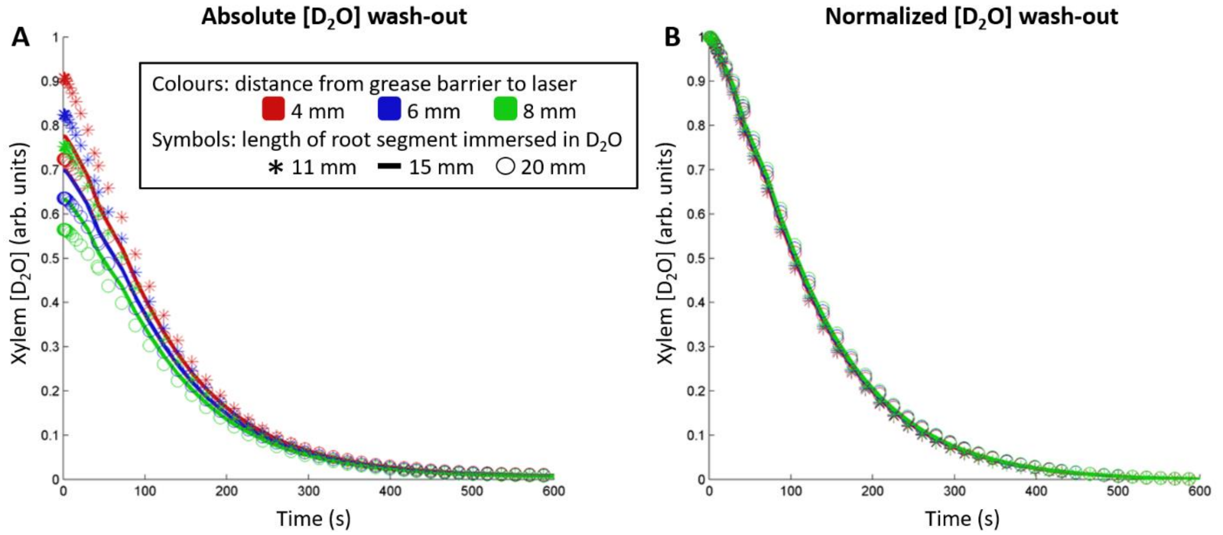

Supplementary Figure 8. Sensitivity analysis of D_2O wash-out traces simulated with MECHA to (i) the length of the distal part of the root previously immersed in D_2O (see symbols in the legend), and (ii) to the distance between the laser focal point and the grease barrier separating the proximal and distal parts of the root (see color code in the legend). A: Absolute fractions of D_2O in xylem water are sensitive to (i) and (ii). B: Normalized fractions of D_2O in xylem water are neither sensitive to factors (i) nor (ii).

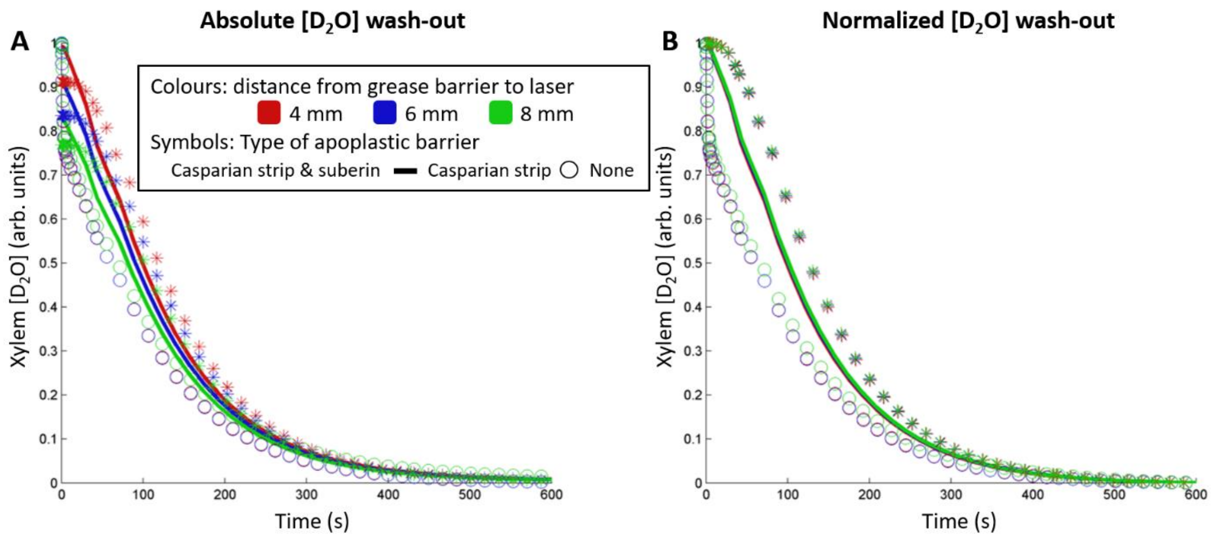

Supplementary Figure 9. Sensitivity analysis of D_2O wash-out traces simulated with MECHA to (i) the type of apoplastic barrier (see symbols in the legend), and (ii) to the distance between the laser focal point and the grease barrier separating the proximal and distal parts of the root (see color code in the legend). A) Absolute fractions of D_2O in xylem water are sensitive to factors (i) and (ii). B) Normalized fractions of D_2O in xylem water remain sensitive to factor (i) only.
