# Supplementary material for: Non-invasive hydrodynamic imaging in plant roots at cellular resolution
Source: Nat Commun. 2021 Aug 3;12:4682. doi: 10.1038/s41467-021-24913-z (PMC8333316; doi:10.1038/s41467-021-24913-z)
Supplement: Supplementary file 3 — Reporting summary [file 41467_2021_24913_MOESM3_ESM.pdf]

nature research

Corresponding author(s): Valentin Couvreur
Flavius C. Pascut

Last updated by author(s): Jul 1, 2021

Reporting Summary

Nature Research wishes to improve the reproducibility of the work that we publish. This form provides structure for consistency and transparency in reporting. For further information on Nature Research policies, see our Editorial Policies and the Editorial Policy Checklist.

Statistics

For all statistical analyses, confirm that the following items are present in the figure legend, table legend, main text, or Methods section.

n/a	Confirmed	
☐	☒	The exact sample size (*n*) for each experimental group/condition, given as a discrete number and unit of measurement
☐	☒	A statement on whether measurements were taken from distinct samples or whether the same sample was measured repeatedly
☐	☒	The statistical test(s) used AND whether they are one- or two-sided<br>*Only common tests should be described solely by name; describe more complex techniques in the Methods section.*
☐	☒	A description of all covariates tested
☒	☐	A description of any assumptions or corrections, such as tests of normality and adjustment for multiple comparisons
☐	☒	A full description of the statistical parameters including central tendency (e.g. means) or other basic estimates (e.g. regression coefficient) AND variation (e.g. standard deviation) or associated estimates of uncertainty (e.g. confidence intervals)
☒	☐	For null hypothesis testing, the test statistic (e.g. *F*, *t*, *r*) with confidence intervals, effect sizes, degrees of freedom and *P* value noted<br>*Give P values as exact values whenever suitable.*
☒	☐	For Bayesian analysis, information on the choice of priors and Markov chain Monte Carlo settings
☒	☐	For hierarchical and complex designs, identification of the appropriate level for tests and full reporting of outcomes
☒	☐	Estimates of effect sizes (e.g. Cohen's *d*, Pearson's *r*), indicating how they were calculated

Our web collection on statistics for biologists contains articles on many of the points above.

Software and code

Policy information about availability of computer code

Data collection

Custom code:
RamanSoftv1_line scan was developed in Visual C# 2008 Express Edition version 9.0.30729.1 SP with Net Framework 3.5 SP1 under Windows XP professional version 5.1.2600 SP3 build 2600.
The custom code is available in the "DataAcquisition" folder under the following publicly available repository:
https://doi.org/10.6084/m9.figshare.14815671
See details in the READ_ME.txt file attached.

Open-access software:
The sofware CellSet (version 1.5.1) was used to collect the root anatomical data required to generate the anatomical layout for MECHA.

Commercial softwares:
1) ANDOR SOLIS + Andor SDK
SOLIS version: 4.2230007.0
SDK version: 2.94.30007.0
ATMCD32CS.dll supplied with the Andor software has to be added to the resources manager inside C# IDE.
2) Prior scientific software Version 1.78.0.0
Interop.PriorLib.dd supplied with the Prior software has to be added to the resources manager inside C# IDE

Data analysis

Custom code:
All the Raman microspectroscopy data was processed in Matlab r2018a with the custom code "DataAnalysis.m".
This custom code is available in the "DataAnalysis" folder under the following publicly available repository:
https://doi.org/10.6084/m9.figshare.14815671
See details in the READ_ME.txt file attached.

The current version of MECHA works in the Anaconda environment for python and was developed on Windows 10 with the IDE Spyder 3.3.6. A Matlab license is required to run the inverse modelling program "MECHA4D_trace_optim.m". Versions dating back to MatlabR2010a have been successfully tested.
The code currently uses the search algorithm fmincon or other algorithms from the Global Optimization toolbox of Matlab.
This custom code is available in the "MECHA_4Dsolute" folder under the following publicly available repositories:
https://doi.org/10.6084/m9.figshare.14892408.v2
https://github.com/MECHARoot/MECHA/blob/master/MECHA_4Dsolute.zip
See details in the READ_ME.txt file attached.

For manuscripts utilizing custom algorithms or software that are central to the research but not yet described in published literature, software must be made available to editors and reviewers. We strongly encourage code deposition in a community repository (e.g. GitHub). See the Nature Research guidelines for submitting code & software for further information.

Data

Policy information about availability of data

All manuscripts must include a data availability statement. This statement should provide the following information, where applicable:

- Accession codes, unique identifiers, or web links for publicly available datasets
- A list of figures that have associated raw data
- A description of any restrictions on data availability

Source data are provided with this paper. The authors declare that the data supporting the findings are openly available online and from the corresponding authors upon reasonable request.
The root hydraulic conductivity data used in this study have been deposited in the FigShare database under accession code https://doi.org/10.6084/m9.figshare.14815911.v2 under CC BY 4.0 licence.
The Raman micro-spectroscopy data generated in this study have been deposited in the FigShare database under accession code https://doi.org/10.6084/m9.figshare.14815971 under CC BY 4.0 licence.
Additional data supporting the findings of this work are available within the paper and its Supplementary Information files.

Field-specific reporting

Please select the one below that is the best fit for your research. If you are not sure, read the appropriate sections before making your selection.

☒ Life sciences ☐ Behavioural & social sciences ☐ Ecological, evolutionary & environmental sciences

For a reference copy of the document with all sections, see nature.com/documents/nr-reporting-summary-flat.pdf

Life sciences study design

All studies must disclose on these points even when the disclosure is negative.

Sample size

Sample size was limited by the amount of time required to conduct the D2O wash-in / wash-out experiments. A 6-month time-limit we imposed for the data acquisition of all plant lines. 10-20 samples for each line was chosen with the expectation that wild type line samples would be measured in the same day as one of the mutant line samples. Hence the number of WT plants measured with Raman is up to twice as many as the individual mutant lines. 3 months were then allocated for data processing with the intention of increasing the sample size if necessary. Based on post-hoc statistical analysis, sample size was large enough to conclude there were highly significant differences (p-value < 0.001) between WT and mutant lines.

Data exclusions

Only plants showing a D2O uptake within the first 10-15min from the sample preparation were selected for the Raman measurements (wash-out, wash-in phase associated to data analysis). This time window was informed by the line-scan experiments presented in Fig 1. Another exclusion criterion was the integrity of the barrier layer; D2O leakage around and under the root observed with Raman near the barrier would indicate a compromised barrier layer.

Replication

Conditions that might affect transpiration (temperature: 20°C, relative humidity: 43%, light levels: 100 µmol.m-2.s-1) and laser power (150 mW, 725 nm) were maintained constant throughout all experiments. Well-characterized Arabidopsis thaliana wild-type (Col-0) and mutant lines (sgn3 myb36, CDEF) at a comparable developmental growth stage were employed. D2O wash-in and wash-out cycles were conducted on sufficient numbers of mutant plants (N=9 and N=18, respectively) to conclude highly significant differences from WT (N=24). Replication sometimes failed due to individual plants not taking up D2O. Such experiments were excluded as described above. Replication was considered successful in all other experiments. For line-scan experiments presented in Fig 1 only a single plant appears, which was subjected to four rounds of intervention (two wash-in and two wash-out cycles). Based on the inter-variability of these data and the kinetics required to be resolved to parameterise the MECHA model, it was determined that subsequent experiments be conducted at a single location (within a protoxylem vessel) - thus following maximum available signal at the maximum possible temporal resolution.

Randomization

The experimenter (FCP) was blind to the nature of the plants examined, with both WT and mutant lines assigned “A”, “B”, “C” etc. Both WT and mutant lines were scanned on every experimental day, in order to block for temporal factors. sgn3 myb36 mutants were acquired later than other plant lines, so were concentrated late in the experimental cycle, but we have no reason to suspect systematic bias since WT responses remained consistent. Potential covariates were controlled by maintaining temperature / RH / light intensity / laser power constant for all experiments as described above. Plant length (root tip to shoot) distribution was chosen to be within the limits of our experimental setup (the size of the microscope 75mm slide with a 15mm Raman silent MgF2 window in the middle) but otherwise randomized with the minimum plant length of 23 mm and maximum length of 44 mm across the entire population (WT and mutants). The chosen Raman interrogation point at a certain distance from the barrier layer was picked between 3mm and 11mm within the constraints of the MgF2 widow and visibility of the xylem vessels under bright field light. It was not possible to shuffle experimenters since Class IV laser use dictated a single highly-trained individual conduct all experiments. FCP designed and constructed the rig, and was the operator throughout. Data analysis

in RMS requires a deep understanding of experimental conditions; hence FCP also conducted the full Raman analysis with no knowledge about the biological identity of individual data sets.

Blinding	The experimenter was blind to the nature of the plants examined, with both WT and mutant lines assigned "A", "B", "C" etc in no particular order. Raman investigator (Flavius C. Pascut) was prevented from knowing the identity of the plant phenotypes. This blinding was maintained throughout experimentation and analysis of each dataset, following which mutant names were assigned to previously-obscured categoricals. No post Raman data analysis was performed after the true identity of each data set was revealed.

Reporting for specific materials, systems and methods

We require information from authors about some types of materials, experimental systems and methods used in many studies. Here, indicate whether each material, system or method listed is relevant to your study. If you are not sure if a list item applies to your research, read the appropriate section before selecting a response.

Materials & experimental systems

n/a	Involved in the study	
☒	☐	Antibodies
☒	☐	Eukaryotic cell lines
☒	☐	Palaeontology and archaeology
☒	☐	Animals and other organisms
☒	☐	Human research participants
☒	☐	Clinical data
☒	☐	Dual use research of concern

Methods

n/a	Involved in the study	
☒	☐	ChIP-seq
☒	☐	Flow cytometry
☒	☐	MRI-based neuroimaging
